# Effect of the Drying Method of Pine and Beech Wood on Fracture Toughness and Shear Yield Stress

**DOI:** 10.3390/ma13204692

**Published:** 2020-10-21

**Authors:** Daniel Chuchala, Jakub Sandak, Kazimierz A. Orlowski, Tomasz Muzinski, Marcin Lackowski, Tomasz Ochrymiuk

**Affiliations:** 1 Department of Manufacturing and Production Engineering, Faculty of Mechanical Engineering, Gdańsk University of Technology, Gabriela Narutowicza Street 11/12, 80233 Gdańsk, Poland; daniel.chuchala@pg.edu.pl (D.C.); kazimierz.orlowski@pg.edu.pl (K.A.O.); 2 InnoRenew CoE, Livade 6, 6310 Izola, Slovenia; jakub.sandak@innorenew.eu; 3 Andrej Marušič Institute, University of Primorska, Muzejski trg 2, 6000 Koper, Slovenia; 4 HS Hydromech, Wybickiego 21, 83050 Lublewo Gdańskie, Poland; tech-rob@wp.pl; 5 The Institute of Fluid-Flow Machinery, Polish Academy of Sciences, Fiszera 14, 80231 Gdansk, Poland; mala@imp.gda.pl

**Keywords:** cutting process, sawing process, cutting power, fracture toughness, drying process, pine wood, beech wood, shear yield stress

## Abstract

The modern wood converting processes consists of several stages and material drying belongs to the most influencing future performances of products. The procedure of drying wood is usually realized between subsequent sawing operations, affecting significantly cutting conditions and general properties of material. An alternative methodology for determination of mechanical properties (fracture toughness and shear yield stress) based on cutting process analysis is presented here. Two wood species (pine and beech) representing soft and hard woods were investigated with respect to four diverse drying methods used in industry. Fracture toughness and shear yield stress were determined directly from the cutting power signal that was recorded while frame sawing. An original procedure for compensation of the wood density variation is proposed to generalize mechanical properties of wood and allow direct comparison between species and drying methods. Noticeable differences of fracture toughness and shear yield stress values were found among all drying techniques and for both species, but only for beech wood the differences were statistically significant. These observations provide a new highlight on the understanding of the effect of thermo-hydro modification of wood on mechanical performance of structures. It can be also highly useful to optimize woodworking machines by properly adjusting cutting power requirements.

## 1. Introduction

Trees contain a high amount of water during their lifetime that is necessary for their survival. Consequently, logs delivered to the sawmill for the downstream conversion are wet, frequently reaching absolute moisture content above 100%, depending on the wood species, season, storing conditions or time after harvesting. Though, a majority of wood products are used in a dry state (10–15%) because of the wood tendency of reaching moisture equilibrium with the surrounding air. Drying of wood is, therefore, an important step in the manufacturing process of any wood product [1]. Several methods of drying wood are commonly used in the modern wood industry with air drying, kiln drying and vacuum drying recognized as the most frequent [1,2,3,4]. Alternative methods using super-critical drying, higher temperature schedules, low pressures, assistance of microwaves, among others, were developed to shorten time of this process [5,6]. Nevertheless, a consequence of each drying event is a change of the native material properties that can occur to a different extent depending on the wood species and process settings—particularly exposure time and treatment temperature.

Herrera-Díaz et al. [7] analyzed an effect of the air temperature during selected drying processes on the mechanical properties of pine wood, including changes to the modulus of elasticity (*MOE*) and modulus of rupture (*MOR*). Two drying methods (air and kiln) as well as thermal modification of radiata pine wood were investigated with a conclusion that values of *MOR* decreased with increase of the process temperatures. Conversely, the *MOE* was unchanged independent of the process type. A similar analysis for spruce wood dried in lower temperatures was performed by Oltean et al. [8]. The result of this study revealed that a significant effect on the mechanical properties (*MOE*, *MOR*, bending strength) was noticed for wood drying temperatures above 80 °C. Roszyk et al. [9] reported that high treatment temperatures of wood affect the *MOE* and *R_c_* (relative/compressive strength) in different directions related to the fibers but do not influence the intrinsic anisotropy of mechanical parameters. Furthermore, Borrega and Kärenlampi [10] noted that the kinetics of the drying process affect an alteration of the mechanical properties of dried wood to the same extent as drying temperature. An effect of the drying method combined with the process temperature on physical properties of dried wood was studied by several researchers [3,11,12,13,14,15]. It was also reported that drying of wood may result in an increase of the material porosity, especially after delignification process, as investigated by Vitas et al. [16]. An effect of the drying method on the parallel compressive strength of bamboo was reported by Arantes et al. [17]. Three methods of drying, including air drying, fire-drying and kiln drying, were compared. A slight reduction of the compressive strength parallel to the fiber direction was detected in the case of the bamboo kiln drying. Generally, the mechanical properties of the wood fall sharply as a result of temperature evenly in all directions, as demonstrated by Fonseca and Barreira [18] and Fonseca et al. [19] on the example of several representative wood species.

Application of a vacuum as an addition to the classical wood drying changes the mechanisms of moisture transport and, consequently, kinetics of the process and resulting material modifications [20,21]. Blanchet et al. [22] reported a small negative effect of the vacuum drying process on the mechanical properties of wood, when compared to conventional oven drying. However, noticeable chemical changes in wood polymers as well as its hygroscopic properties because of high-temperature heat treatment were reported by several researchers [23,24,25,26]. These chemical changes are expected, therefore, to affect other material characteristics including strength and mechanical properties. Indeed, a gradual reduction of poplar’s mechanical strength was observed by Sandak et al. [27] when increasing the vacuum thermal treatment time, temperature and, consequently, the process intensity. An elevated temperature of wood drying, and its thermal modification, resulted in the higher fragmentation and granularity of sawdust obtained during sawing, milling or sanding processes [28,29,30,31]. This indicates an extensive change in the fracture properties of such treated wood. 

It can be concluded, therefore, that diverse drying methods affect (to varying degrees) the mechanical, physical and chemical properties of dried wood. It is a common understanding that rapid reduction of dynamic mechanical properties is the first and most noticeable consequence of drying or thermal treatment. It is related to increased brittleness of the resulting materials. Hence, it can be hypothesized that drying affects the related mechanical properties, particularly fracture toughness *R* and shear yield stress *τ_γ_*. Both properties are relatively difficult to be estimated in routine material characterization tests but are highly relevant for the engineered structure safety assurance as defined in building codes. *R* and *τ_γ_* are also fundamental for the proper estimation of cutting forces and corresponding cutting power. Optimization of cutting processes is highly relevant when considering the overall economy of production and manufacturing process in woodworking and other sectors. An accurate forecasting of the power demand for the cutting of wood allows optimal choice of the process configuration, including the number of saws and their spacing in frame saws, multi-circulars saws or tandem band saws. This ensures an effective use of machines as well as prevents overloading that can result in the damage of tools or other losses associated with long downtime in the manufacturing process [32,33,34]. 

A consistent model of cutting power requirements based on mechanical properties of wood (both fracture toughness *R* and shear yield stress *τ_γ_*) has been proposed in the proceeding works [35,36,37,38]. This model is comprehensive for diverse cutting configurations and can be used to predict the cutting forces acting on different process kinematics or tools of various geometries [38,39,40,41]. 

The goal of this study was to determine variations within fracture toughness and shear yield stresses of hard and soft woods due to differences in the applied drying procedures. The mentioned material properties could be useful for engineers designing wooden structures as well as for the proper estimation of the cutting power requirements. 

## 2. Materials and Methods

### 2.1. Materials

Scots pine (*Pinus sylvestris* L.) and beech (*Fagus silvatica* L.) species were used for preparation of experimental samples. The green wood, originated from Pomeranian District of Poland, was not exposed to any intended drying processes nor thermal treatment before preparation of experimental samples. Boards from the sawmill were exposed to four different drying processes following standard industrial protocols as well as laboratory prototypes. Ten blocks with dimensions ***W*** = 60 mm × ***H_p_*** = 60 mm × ***L_p_*** = 700 mm (width×height×length, respectively) were cut-out from randomly selected boards. The wood density, considered here as a ratio of air-dry wood mass to its volume, was measured separately on each block and results are summarized in Table 1. 

The moisture content MC of wood was determined with resistance-based moisture content meter WRD 100 (TANEL SJ, Gliwice, Poland) with declared accuracy of ±2%. The drying of wood was performed in four alternative scenarios, with measured initial moisture content MC = 40% for pine and MC = 70% for beech. 

**Figure 1:**
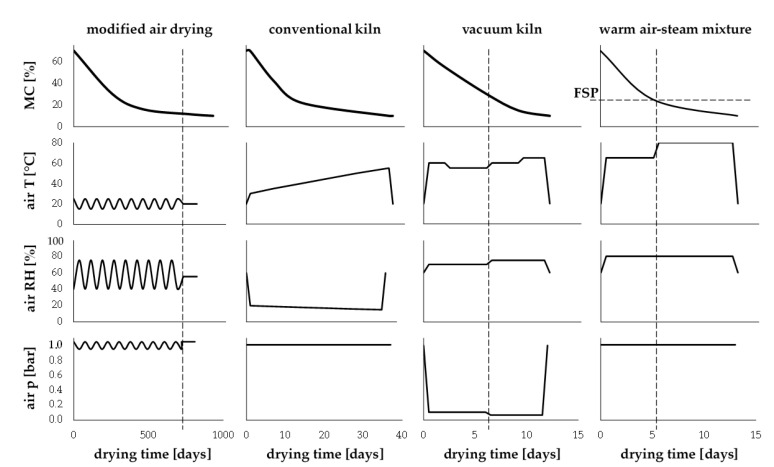
Schematic of schedules for dying experimental beech samples with four studied processes. Note: air p—air pressure, air RH—air relative humidity, air T—air temperature, FSP—fiber saturation point.

A schematic representation of four drying process configurations is summarized in Figure 1 and most relevant technical details are described below. 

#### 2.1.1. Modified Air-Drying Process

The first method for investigation of the changes to the wood induced by drying was a classic air-drying process [1]. In that case, the batch of green wood samples was stored for two years, outdoors under a roof, at the Gdansk University of Technology campus, assuring proper ventilation and shadowing. After initial phase of natural drying, the wood moisture content reached approximately MC = 16% for both species. In order to reduce the MC to the usual level of indoor use, all the samples were additionally conditioned in the laboratory conditions assuring constant air temperature of 20 °C and relative humidity of 55%. The second phase of air drying took three months, and the resulting MC was reduced to 10%. Such level of MC was assumed as similar to that obtained when drying wood according to state-of-the-art industrial solutions.

#### 2.1.2. Conventional Kiln Drying Process

The second batch of experimental samples was dried in the industrial kiln O.S Panto 120/F (PANTO, Szczytno, Poland) installed at the wood processing company PHU Drew-Met from Kiełpino, Poland. Experimental samples were mixed with a similar batch of wood that was used for the production needs of the factory. The drying process implemented followed the routine drying schedules of the company in varying temperatures of 30 °C to 55 °C for beech and 35 °C to 75 °C for pine. The drying process control assured adaptation of air parameters following MC changes of processed wood. It took 14 and 37 days to dry pine and beech samples, respectively. It is a standard procedure implemented in the PHU Drew-Met company to cool down the kiln and condition the wood before the conclusion of the drying process. The final moisture content of both investigated woods was MC = 10%.

#### 2.1.3. Vacuum Kiln Drying Process

An alternative to classic kiln drying, widely implemented in the wood industry, is vacuum kiln drying that was also tested in this research. The third batch of experimental samples was processed at the same company as kiln drying (PHU Drew-Met, Kiełpino, Poland) using the industrial vacuum kiln SP-5 (LAC S.R.O., Židlochovice, Czech Republic). The wood was heated by direct contact with hot plates and conduction [3]. The drying schedule followed routine settings of the company. The vacuum inside of the kiln was 100 mbar (10 kPa) with temperature of 65 °C for 88 h when drying pine wood. The process became two-steps in the case of beech with the initial pre-drying phase of 55 °C at 10 kPa (84 h), followed by the final drying phase of 65 °C at 6 kPa for 65 h.

#### 2.1.4. Warm Air–Steam Mixture Experimental Drying Process

The fourth batch of samples was dried in the experimental kiln developed at the Gdansk University of Technology (GUT). The technical details regarding that innovative process are presented in the work of Baranski [42]. The method uses heated steam as a drying medium and resulting materials are considered as a hybrid thermally treated wood. The specific settings of the drying schedule were adopted taking into consideration available recommendations [43,44]. The process consisted of two-stages where temperature of the medium in the kiln increased from 65 °C (stage one) to 80 °C at the second stage. The first stage lasted until the wood reached fiber saturation point *FSP*. In the case of pine, it was approximately 32 h, and for beech 125 h. Total time of drying process for pine was approximately 90 h and in the case of beech 300 h. The relative humidity *RH* of the heated steam was constant along the process (*RH* = 80%), while the drying medium flow velocity was 2.5 m∙s^−1^. The whole batch of wooden boards was cooled down before opening the kiln to reduce the induced stresses.

### 2.2. Machinability Tests

Experimental cuttings were performed on the PRW15M sash gang saw with a hybrid dynamically balanced driving system and elliptical teeth trajectory movement. The concept of the machine was developed at Gdańsk University of Technology [45] and prototype manufactured by REMA S.A. (Reszel, Poland). The use of electric power (active and passive) during idling and working cycles was continuously monitored with the power converter PP54 (LUMEL S.A., Zielona Góra, Poland). The data were recorded with a time stamp and further processed to determine energetic effects of cutting. A detailed list of sawing machine settings and used tool characteristics is summarized in Table 2. 

The mean value of feed per tooth *f_z_* for a sash gang saw was calculated as in Equations (1) and (2) [31,41]:

(1) $$f_{z}=\frac{1000\cdot v_{f}\cdot t_{p}}{n_{F}\cdot H_{F}}$$

(2) $$v_{f}=\frac{L_{p}}{t_{c}}$$

where: *v_f_*—feed speed (m·min^−1^), *t_p_*—tooth pitch (mm), *L_p_*—length of the sample (m), *H_F_*—saw frame stroke (mm), *n_F_*—number of strokes of saw frame per min (spm) and *t_c_*—cutting time (min) necessary to process sample of the length *L_p_*.

The average cutting power *P_c_* was calculated as the difference of the mean total power *P_T_* and the average idle power *P_i_* [41,46], as expressed in Equation (3):

(3) $$P_{c}=P_{T}-P_{i},$$

The average idle power *P_i_* of the frame saw PRW15-M was determined each time before initiation of the proper cutting cycle. It allowed minimization of an effect of the varying temperature of the machine components (such as hydraulic oil, gear boxes, etc.) on the energetic effects corresponding directly to the cutting process. The average cutting power in a working stroke *P_cw_* was calculated as in Equation (4), following the works [41,46]:

(4) $$P_{cw}=2\cdot P_{c},$$

### 2.3. Methodology for Determination of Material Properties from the Cutting Test

The average total cutting power in the working stroke *P_cT_* for a single saw blade in the sash gang can be determined by means of the cutting forces model proposed by Atkins [47,48]. This methodology was adopted for the case of cutting wood on the frame sawing machine by authors Orlowski et al. [35], Chuchala et al. [39] and Sinn et al. [41] and is summarized in Equation (5):

(5) $$P_{cT}=P_{cw}+P_{ac}+P_{dull}=\underset{❶}{\underbrace{\frac{m\cdot H_{p}\cdot \tau_{\gamma }\cdot \gamma \cdot S_{t}}{Q\cdot t_{p}}h\cdot v_{c}}}+\underset{❷}{\underbrace{\frac{m\cdot H_{p}\cdot R\cdot S_{t}}{Q\cdot t_{p}}\cdot v_{c}}}+\underset{❸}{\underbrace{P_{ac}}}+\underset{❹}{\underbrace{P_{dull}}},$$

Equation (5) consists of four components. The first term ❶ describes the internal work of plasticity along the shear plane, where *τ_γ_* is shear yield stress and *γ* is the shear strain along the shear plane. The value of γ can be calculated according to Equation (6), assuming that Φ*_c_* corresponds to the shear angle:

(6) $$\gamma =\frac{\cos\gamma_{f}}{\cos\left(\Phi_{c}-\gamma_{f}\right)\cdot \sin\Phi_{c}},$$

where: *γ_f_*—tool side rake angle.

The second component ❷ defines internal work required for the formation of new surface, where *R* corresponds to the fracture toughness or specific work of material separation. The coefficient of friction correction *Q* appears in both ❶ and ❷, representing an effect of friction between tool rake face and separated material. *Q* is computed according to Atkins [47,48] and Orlowski et al. [35] and can be represented as in Equation (7):

(7) $$Q=1-\frac{\sin\beta_{\mu }\cdot \sin\Phi_{c}}{\cos(\beta_{\mu }-\gamma_{f})\cdot \cos(\Phi_{c}-\gamma_{f})},$$

where: *β_μ_* = tan^−1^*μ* is a friction angle (rad) directly related to the coefficient of friction *μ*.

The other parameters included in the first two terms of Equation (5) are as follow: *m*—number of saws in the gang, *H_p_*—height of cutting material, *S_t_*—overall set (kerf width), *h*—uncut chip thickness, *v_c_*—cutting speed.

The power needed for acceleration of chips *P_ac_* is also included in Equation (5) as component ❸. It can be described as variation function of the mass flow and tool velocity [35]. It is important to notice that the value of chip acceleration power *P_ac_* is calculated globally for the sawing process and is not directly related to the number of working teeth. As the contribution of *P_ac_* on the overall cutting power *P_cT_* is negligible [41,46], it is not considered in analyses performed for the needs of this research.

The last component ❹ of Equation (5) corresponds to the excessive energy use in case of improper chip formation related to the dullness of the cutting edge. It is an important component of the energetic balance of the real-world processes, and it explains an increase of the cutting forces observed along the tool life and increase of dullness. However, assuring an appropriate sharpness of the tool, component ❹ can be ignored, especially at the initial phase of the tool use. Summarizing, the following assumptions were made for the needs of this research:

- only freshly sharpened blades were used in cutting tests: component ❹ of Equation (5) = 0;
- chip acceleration power *P_ac_* is omitted: component ❸ of Equation (5) = 0;
- values of the shear angle Φ*_c_* were calculated following the approach of Merchant [46], considering specific cutting zone geometry as well as coefficient of friction. This procedure is valid when deviations in the shear angle assigned to inherent material properties can be neglected for larger values of uncut chip thicknesses, as used in this experiment;
- the value of friction coefficient *µ*= 0.6 was adopted following the work of Glass and Zielinka [49];
- the effect of lateral forces on the power consumption can be omitted when cutting in straight direction and with minimal saw deviation [50].

As a consequence, it is possible (by implementing the above listed assumptions) to express Equation (5) as a linear regression function (Equation (8)):

*P_cw_*(*h*) = *c*_1_ ∙ *h* + *c*_0_, (8)

**Figure 2:**
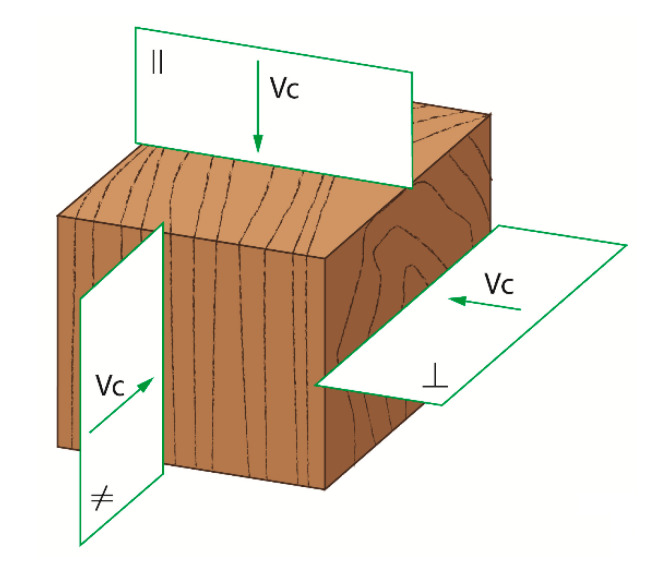
Cutting speed directions when splitting orthotropic materials; axial || cutting along fibers (cutting direction 90°–0°), perpedicular ⊥ cutting across fibers (cutting direction 90°–90°), and cross-cutting ≠ direction (cutting direction 0°–90°) [38,52,53].

In that case, *c*_1_ and *c*_0_ correspond to the slope and intercept, respectively. An independent variable of the regression is the uncut chip thickness *h.* It has become possible, therefore, to determine values of fracture toughness *R*_⊥_ and shear yield stress *τ_γ_*_⊥_ by matching the regression Equation (8) with the experimental data from the cutting tests. A similar approach was reported for diverse materials and cutting kinematics [40,46,51]. However, in the case of the frame sawing process investigated here, values of both fracture toughness *R*_⊥_ and shear yield stresses *τ_γ_*_⊥_ are determined for cutting perpendicular to wood fibers direction Φ*_G–vc_* (case 90−90 according to Kivimaa [52]). The detailed cutting configuration is presented in Figure 2.

The mathematical procedure for computation of above material properties is expressed in Equations (9) and (10) for *τ_γ_*_⊥_ and *R*_⊥_, respectively:

(9) $$\tau_{\gamma ⟂}=\frac{c_{1}\cdot Q}{z_{a}\cdot \gamma \cdot S_{t}\cdot v_{c}},$$

(10) $$R_{⟂}=\frac{c_{0}\cdot Q}{z_{a}\cdot S_{t}\cdot v_{c}},$$

where *z_a_* = *H_p_*/*t_p_* – number of teeth in contact with the kerf (average).

## 3. Results and Discussion

**Figure 3:**
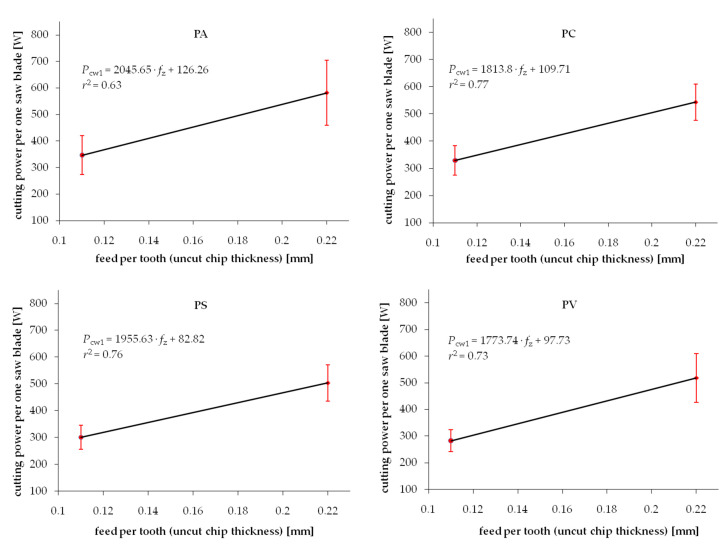
Relation between cutting power per one saw blade and uncut chip thickness when sawing pine wood dried with different methods: PA—air drying, PC—conventional kiln drying, PS—warm heated-steam experimental drying, PV—vacuum kiln drying.

**Figure 4:**
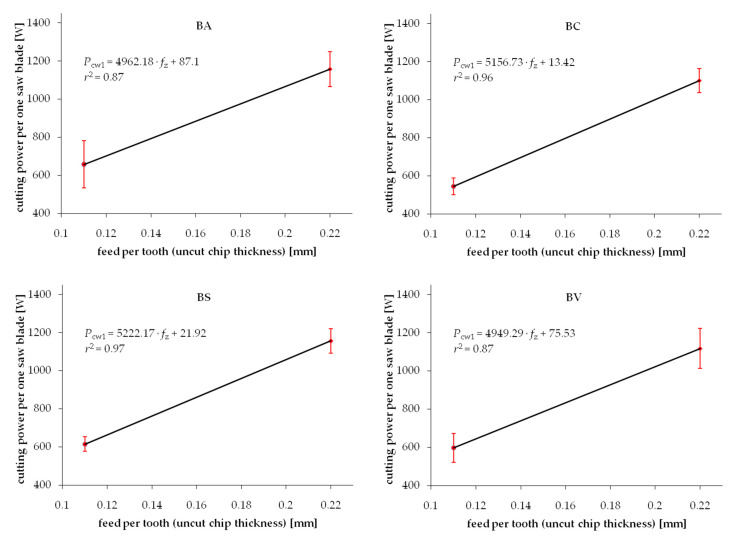
Relation between cutting power per one saw blade and uncut chip thickness when sawing beech wood dried with different methods: BA—air drying, BS—warm heated-steam experimental drying, BC—conventional kiln drying, BV—vacuum kiln drying.

Experimental results from the series of cuttings performed on wood samples exposed to different drying procedures are summarized in Figure 3 and Figure 4 for Scots pine and beech, respectively. Each chart presents two test point groups that correspond to the mean value and standard deviations of measured cutting powers at two levels of feed speed *v_f_*. Values of feed speed corresponds to the basic geometrical parameter of the cutting process, i.e., uncut chip thickness *h*. The experimental results are clustered around values *h* = 0.11 mm and *h* = 0.22 mm. However, exact values of the feed per tooth (that correspond to *h* in the case of such gang saws) were determined individually for each processed sample based on recorded experimental data. The data fitting curve (linear regression), as well as regression equation with coefficient *c*_1_ and intercept *c*_0_, are provided in each chart. A relatively wide range of standard deviations noticed for similar values of feed speed can be observed in both figures. It is related to the high variance of chemical-physical properties native to biomaterials. In the case of experimental wood samples, it was associated to the within batch differences of wood density as well as change of mechanical properties induced by the drying process. Furthermore, the density variation within a single sample, which is associated with the early and late wood differences, presence of wood defects or other common irregularities of the wood tissue, are recorded during cutting tests, increasing even more the scatter of results. Even if all the care was given to assure a homogenous and defect-less set of samples used for different drying experiments, the average density would vary between samples (Table 1). Standard deviation ranges of density within batches in individual test groups corresponding to studied drying methods are summarized in Table 1.

The evident effect of wood density *ρ* [54,55] can be compensated by normalizing values of resulting mechanical parameters [41]. Diverse algorithms can be implemented for the data unification and further non-biased interpretation. The approach adopted for the need of this study included correction of the measured cutting power by the variation related to the average wood assessed individually for each processed board. Consequently, Equation (5) was revised as follows (Equation (11)), assuming that *P_ac_* and *P_dull_* can be neglected for the cutting process on the frame saw:

(11) $$P_{cT}=P_{cw}=\frac{m\cdot H_{p}\cdot S_{t}}{Q\cdot t_{p}}\cdot (\tau_{{}_{\gamma }}^{*}\cdot \gamma \cdot h+R^{*})\cdot v_{c}\cdot \rho ,$$

Two novel meta variables were introduced here that correspond to normalized fracture toughness R^*^_⊥_ [J m kg^−1^] and normalized shear yield stress in the shear zone τ^*^_γ__⊥_ [MPa m^3^ kg^−1^]. Equation (11) can, therefore, be expressed as a lineal equation by following the same logic as used for deriving Equation (8). In that case, the cutting power related to the wood density as a function of the chip thickness h is presented in Equation (12):

(12) $$P_{cw}{}^{*}(h)=\frac{P_{cw}}{\rho }(h)=c_{1}\cdot h+c_{0},$$

**Figure 5:**
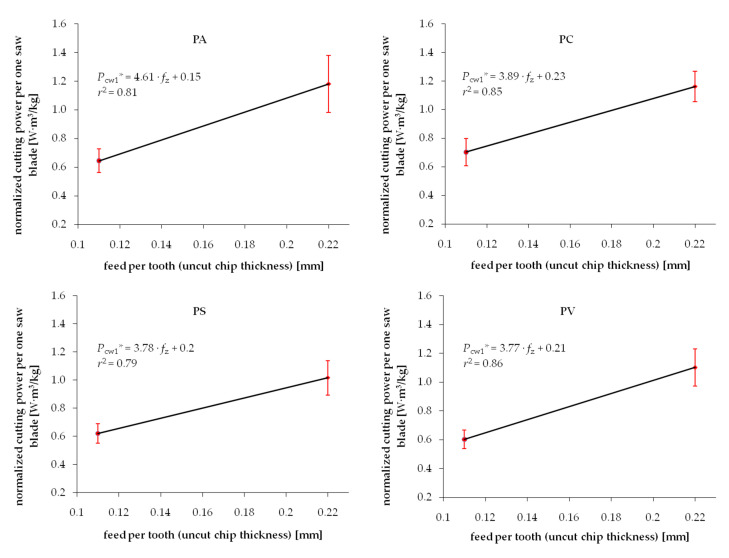
Relation between normalized (by density) cutting power per one saw blade and uncut chip thickness when sawing pine wood dried with different methods: PA—air drying, PC—conventional kiln drying, PS—warm heated-steam experimental drying, PV—vacuum kiln drying.

**Figure 6:**
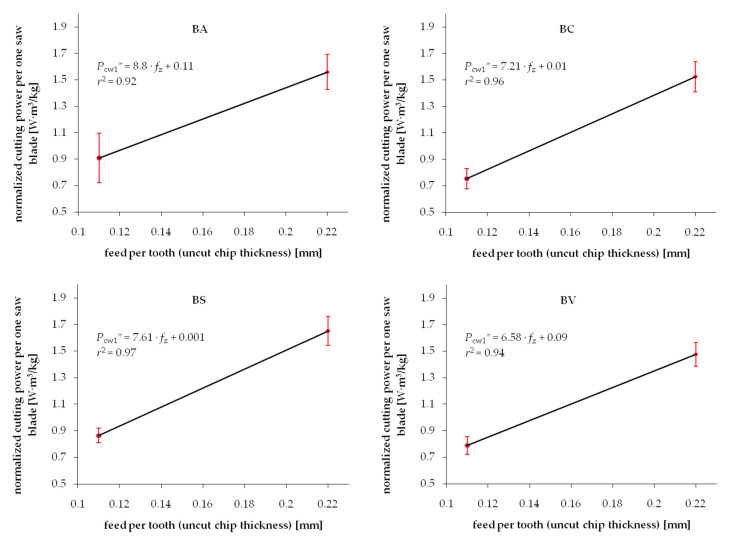
Relation between normalized (by density) cutting power per one saw blade and uncut chip thickness when sawing beech wood dried with different methods: BA—air drying, BC—conventional kiln drying, BS—warm heated-steam experimental drying, BV—vacuum kiln drying.

The experimental values of the cutting power per one saw blade P_cw_* normalized by density are shown in Figure 5 and Figure 6 for pine and beech, respectively. It is evident that the standard deviation ranges are noticeably reduced compared to the not-normalized results. It is confirmed by higher values of determination coefficients *r*^2^ in the majority of investigated cases. This phenomenon confirms the hypothesis of the direct correlation of the density and fracture properties of wood. However, it is also clear that considering density only does not lead to complete elimination of the experimental data scatter. It was previously observed by Chuchala et al. [56] that the effect of density upon the cutting power (cutting forces) is better explained if also considering the synergetic effect of varying cutting conditions, such as feed speed. It is clearly visible in Figure 3, Figure 4, Figure 5 and Figure 6 that the repeatability of results, observed as a minimized standard deviation ranges, is higher for low chip thicknesses.

Average values of R^*^_⊥_ and τ^*^_γ__⊥_ for Scots pine are summarized in Table 3 together with standard deviations of the observed variation. The fracture toughness increases when wood is dried in other than air drying conditions. The air drying [1] is considered as a reference method that affects the native physical and chemical properties of wood to a small extent [2]. That leads to the conclusion that industrially dried wood becomes more brittle than wood not exposed to elevated temperatures. Conversely, the shear yield stress slightly decreases, making the Scots pine wood easier to deform. A heated-steam experimental drying appears to be the mildest as the fracture parameters were less altered. The relatively high range of the observed values variation is affected by rather low number of cutting tests implemented in this pilot research. Statistical analyses of the experimental results are summarized in Table 4. No significant differences were noticed in the case of pine wood for both the normalized fracture toughness R^*^_⊥_ and normalized shear yield stress τ^*^_γ__⊥_.

A very diverse effect of investigated drying techniques on the fracture properties of hardwood (beech) can be derived from data presented in Table 3. While the average shear yield stress (normalized by the wood density) was not large affected by different drying techniques, the fracture toughness evidently dropped when drying in industrial kilns. It is especially noticeable for wood dried according to experimental heat stem approach where R^*^_⊥_ reached negligible values. The wood of beech consists of a significant share of hemicelluloses that are very sensitive to thermo-hydro modification. It results in extensive hydrolysis when exposed to elevated temperatures in the presence of moisture. Even if the mass loss due to mild thermal modification during drying is negligible, the chemical reconfiguration of hemicelluloses, evidenced as a decrease of hygroscopicity, was observed [15,25,27,57]. It was also reported that thermolysis of hemicelluloses is highly inhibited when drying/thermal modification occurs in vacuum [25,27]. This was confirmed in this research where fracture toughness of vacuum dried wood decreased only slightly. The ANOVA test revealed significant differences between the determined normalized fracture toughness and normalized shear yield stress values for beech wood dried by four different methods (Table 4). However, no statistical differences were detected between air-dried and vacuum-dried beech wood.

## 4. Conclusions

The research presented allows us to derive the following conclusions:

- The wood machining process on the frame saws can be adopted for straightforward determination of the fracture properties of materials by monitoring the power needed for cutting.
- Wood density is an important factor affecting the mechanism of the chip formation and should, therefore, be considered when interpreting cutting test results. A straightforward way for the results normalization was presented in this report.
- Technique of wood drying affects the physical-chemical properties that are revealed as a change of the fracture toughness and shear yield stress derived from the cutting tests.
- The observed trend of fracture properties changes was different for examined softwood (Scots pine) and hardwood (beech). Statistically significant differences (α = 0.05) between the determined mechanical properties of wood dried by four different methods were observed only for beech. It is related to differences in microstructure of both species and diverse deterioration mechanisms when exposing wood to elevated temperatures.
- The methodology presented here can be highly useful for accurate forecasting of the cutting power that is indispensable to optimize construction of woodworking machines and tools.

The results obtained revealed that drying wood with the use of high temperature air-steam mixture significantly reduces the fracture toughness of beech wood. However, it was not noticed for pine wood. Further studies and experimentation are therefore indispensable to properly understand mechanisms of material changes due to drying.

## Figures and Tables

**Table 1:** Density of tested pine and beech wood samples.

Sample Code	Drying Method	Wood Density *ρ* (Mean Value and Standard Deviation) [kg·m^−3^]
	Pine Wood	
PA	modified air drying	515 ± 57.1
PC	conventional kiln drying	467 ± 26.4
PS	heated-steam experimental drying	490 ± 33.5
PV	vacuum kiln drying	474 ± 41.5
	**Beech Wood**	
BA	modified air drying	723 ± 52.0
BC	conventional kiln drying	722 ± 36.9
BS	heated-steam experimental drying	706 ± 25.4
BV	vacuum kiln drying	757 ± 38.5

**Table 2:** Settings of the sash-gang saw, and its saw blade as used in the experimental cuttings.

Parameter		Symbol	Value	Unit
**Machine Setting**				
number of strokes of saw frame per min		*n_F_*	685	spm
saw frame stroke		*H_F_*	162	mm
number of saws in the gang		*m*	5	–
average cutting speed		*v_c_*	3.69	m·s^−1^
feed speed	slow	*v*_*f*1_	0.9	m·min^−1^
	fast	*v*_*f*2_	1.9	m·min^−1^
feed per tooth	slow	*f*_*z*1_	0.11	mm
	fast	*f*_*z*2_	0.22	mm
**Tool Setting**				
the sharp saw blades with stellate tipped teeth		–	–	–
overall set (kerf width)		*S_t_*	2	mm
saw blade thickness		*s*	0.9	mm
free length of the saw blade		*L*_0_	318	mm
blade width		*b*	30	mm
tooth pitch		*t_p_*	13	mm
tool side rake angle		*γ_f_*	9	°
tool side clearance		*α_f_*	14	°
tension stresses of saws in the gang		*σ_N_*	300	MPa

**Table 3:** Fracture toughness R^*^_⊥_ and shear yield stress τ^*^_γ__⊥_ normalized by density (average value and standard deviations) for of Scots pine and beech dried with different methods.

Sample Code	Drying Method	Normalized Fracture Toughness R^*^_⊥_ [J·m·kg^−1^]	Normalized Shear Yield Stress τ^*^_γ__⊥_ [MPa·m^3^·kg^−1^]
**Pine Wood**			
PA	air drying	2.72 (±1.66)	0.040 (±0.009)
PC	conventional kiln drying	3.69 (±1.54)	0.036 (±0.007)
PS	heated-steam experimental drying	3.34 (±1.20)	0.032 (±0.006)
PV	vacuum kiln drying	3.59 (±1.08)	0.034 (±0.006)
**Beech Wood**			
BA	air drying	2.42 (±0.65)	0.062 (±0.003)
BC	conventional kiln drying	1.08 (±0.40)	0.066 (±0.003)
BS	heated-steam experimental drying	0.39 (±0.21)	0.068 (±0.002)
BV	vacuum kiln drying	1.70 (±1.03)	0.064 (±0.003)

**Table 4:** Significance of differences between fracture toughness and shear yield stress of pine and beech samples dried with different methods (ANOVA) (α = 0.05).

Pine Wood							
Sample Code	Source	DF	Adj SS	Adj MS	F-Value	*p*-Value	F-Critical
Fracture Toughness							
PA	between groups	3	4.1858	1.3953	0.7347	0.5404	2.9604
PC	within groups	27	51.2738	1.8990			
PS	total	30	55.4596				
PV							
Shear Yield Stress							
PA	between groups	3	0.000285	9.51 × 10^−5^	1.9436	0.1464	2.9604
PC	within groups	27	0.001321	4.89 × 10^−5^			
PS	total	30	0.001606				
PV							
**Beech Wood**							
Fracture Toughness							
BA	between groups	3	14.2889	4.7630	9.6225	0.00026	3.0280
BC	within groups	23	11.3845	0.4950			
BS	total	26	25.6734				
BV							
Shear Yield Stress							
BA	between groups	3	0.000134	4.46 × 10^−5^	3.1743	0.04333	3.0280
BC	within groups	23	0.000323	1.4 × 10^−5^			
BS	total	26	0.000457				
BV							

## List of Symbols

*BA* *BC* *BV* *BS*	sample codes for beech wood	*S_t_*	overall set (kerf width)
*FSP*	fiber saturation point	*T*	air temperature
*H_F_*	saw frame stroke	*W*	sample width
*H_p_*	sample height	*c*_0_, *c*_1_	coefficients of linear regression function
*L_p_*	sample length	*f_z_*	feed per tooth
*MC*	moisture content	*h*	uncut chip thickness
*MOE*	modulus of elasticity	*m*	saws number
*MOR*	modulus of rupture	*n_F_*	number of strokes of saw frame per min
*P_ac_*	power needed for acceleration of chips	*p*	air pressure
*P_c_*	average cutting power	*t_c_*	cutting time
*P_cT_*	overall cutting power	*t_p_*	tooth pitch
*P_cw_*	average cutting power in a working stroke	*v_c_*	cutting speed
*P*_cw_*	average cutting power in a working stroke normalized by density	*v_f_*	feed speed
*P_dull_*	power needed to chip formation while dullness of the cutting edge	*z_a_*	number of teeth in contact with the kerf (average)
*P_i_*	average idle power	*β_μ_*	friction angle
*P_T_*	mean total power	*γ*	shear strain along the shear plane
*PA* *PC* *PV* *PS*	sample codes for pine wood	*γ_f_*	rake angle
*Q*	coefficient of friction correction	*μ*	friction coefficient
*R*	fracture toughness	*ρ*	density
*R*_⊥_	fracture toughness in perpendicular direction	*τ_γ_*	shear yield stress
*R*_⊥_*	fracture toughness in perpendicular direction normalized by density	*τ_γ_*_⊥_	shear yield stress in perpendicular direction
*R_c_*	relative/compressive strength	*τ***_γ_*_⊥_	shear yield stress in perpendicular direction normalized by density
*RH*	relative humidity	Φ*_c_*	shear angle
